# DiColorimetry: A smartphone application for colorimetric analysis of phenolics and antioxidant activity in vegetable oils

**DOI:** 10.1016/j.mex.2026.104144

**Published:** 2026-09-04

**Authors:** Sanita Vucane, Ingmars Cinkmanis, Martins Sabovics, Heinrihs Cinkmanis

**Affiliations:** a Food Institute, Faculty of Agriculture and Food Technology, Latvia University of Life Sciences and Technologies, Lielā Street 2, Jelgava, LV-3001, Latvia; b Jelgava Technical School, Pulkveža Oskara Kalpaka Street 37, Jelgava, LV-3001, Latvia

**Keywords:** Smartphone-based colorimetry, Digital image analysis, DiColorimetry, Total phenolic content, Radical scavenging activity, Vegetable oils, Android application

## Abstract

This method presents DiColorimetry, an author-developed Android application for smartphone-assisted colorimetric assessment of total phenolic content (TPC), radical scavenging activity (RSA) and general colour characteristics in vegetable oils. Smartphone-based imaging itself is not presented as a new analytical principle; the methodological contribution is the integration of previously separate image acquisition, ROI-based colour extraction, assay-specific signal processing, calibration or reference handling, calculation and data export within a single mobile workflow.

• The application integrates assay-specific TPC and RSA analysis with a standalone colour-data extraction module, including region-of-interest selection and CSV export.
• Analytical performance was evaluated using 40 commercial vegetable oil samples from ten botanical categories and compared with UV/VIS spectrophotometry under controlled imaging conditions using a Samsung Galaxy A33 5 G smartphone.
• DiColorimetry showed close agreement with the reference method: mean signal-acquisition relative standard deviations were below 1%, and all paired TPC and RSA results showed absolute relative deviations below 5%.
• The standalone module provides RGB, HSV, CIELAB, and CIELCh values but requires a user-established calibration model for quantitative application. DiColorimetry is intended as an integrated assay-specific smartphone readout and screening tool rather than as a new smartphone-imaging principle or a universal replacement for calibrated analytical instrumentation.

## Graphical abstract

**Figure:**
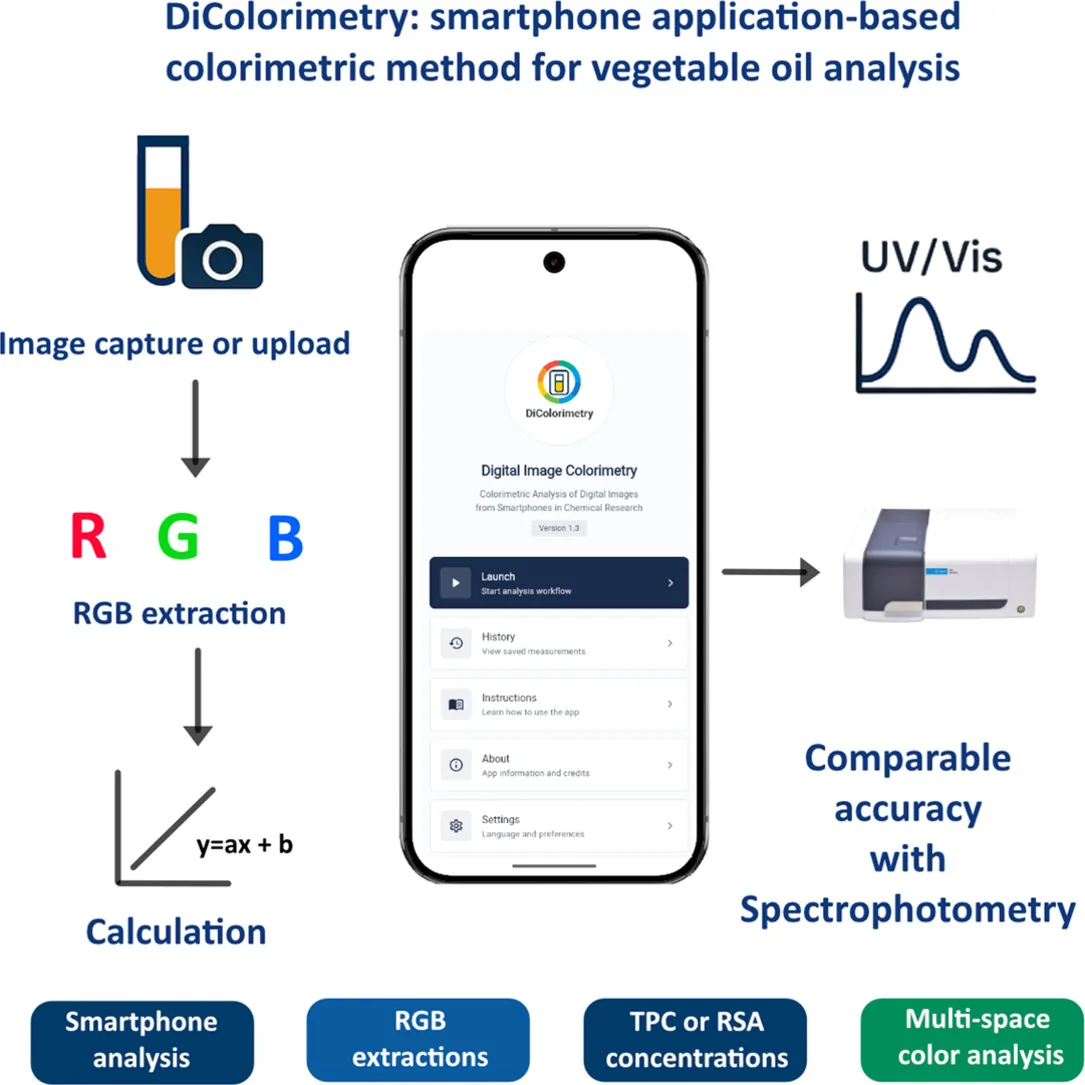

Specifications table

**Table:** 

**Subject area**	Chemistry
**More specific subject area**	Analytical chemistry; food analysis; smartphone-based digital image colorimetry
**Name of your method**	DiColorimetry-assisted smartphone digital image colorimetry for total phenolic content and radical scavenging activity determination in vegetable oils
**Name and reference of original method**	S. Vucane, I. Cinkmanis, K. Juhnevica-Radenkova, M. Sabovics, Revolutionizing phenolic content determination in vegetable oils: a cutting-edge approach using smartphone-based image analysis, Foods 13 (2024) 1700, https://doi.org/10.3390/foods13111700; S. Vucane, M. Sabovics, L. Leitans, I. Cinkmanis, Smartphone-based colorimetric determination of DPPH free radical scavenging activity in vegetable oils, Research for Rural Development 35 (2020) 106–111, https://doi.org/10.22616/rrd.26.2020.016
**Resource availability**	The DiColorimetry Android application (version 1.2), APK file, user documentation and technical metadata are available via Zenodo: https://doi.org/10.5281/zenodo.17819093 . Sample information and additional channel-comparison data are provided in the Supplementary Material. Reagents, equipment, and standardized imaging conditions are described in the Method details section.

## Background

Classical instrumental methods are widely used for the quantitative analysis of bioactive compounds in food and plant-based matrices. Although these methods provide reliable and sensitive analytical data, their routine application often requires specialized laboratory equipment, controlled working conditions, and trained personnel [[1], [2], [3]]. These requirements may limit their use in teaching laboratories, small research facilities and situations where rapid, portable, or low-cost screening is needed.

Smartphone-based digital image colorimetry has emerged as a practical alternative for colorimetric analysis when image acquisition conditions are standardized [[4], [5], [6], [7], [8], [9]]. In such approaches, a smartphone camera is used to capture the color response of a sample, while image-processing tools extract color information that can be related to analyte concentration or antioxidant-related activity. The RGB color model has been widely used in smartphone-assisted chemical analysis because it provides a simple and accessible way to quantify color intensity changes [10]. Previous studies have demonstrated the applicability of smartphone- or scanner-based image analysis for total phenolic content determination and antioxidant activity assessment in plant-based products [[11], [12], [13], [14], [15], [16], [17], [18]].

Vegetable oils contain phenolic compounds and other antioxidant-related constituents that contribute to their nutritional, functional and quality-related properties [[19], [20], [21]]. Smartphone-based image analysis has also been explored for vegetable oil authentication, classification, fluorescence-based identification, shelf-life monitoring and iodine value determination [7,[22], [23], [24], [25]]. However, the literature still contains limited examples of dedicated mobile applications designed specifically for quantitative colorimetric analysis of vegetable oils.

Many reported smartphone workflows require separate image-processing applications, scripts, calibration tools, or external calculation steps. In the authors’ previous studies, TPC determination was implemented using a Python-based image-processing algorithm, whereas DPPH radical scavenging activity was assessed using a separate colour-picking application [26,27]. This fragmentation motivated the development of DiColorimetry, an author-developed Android application that integrates image capture or upload, manual region-of-interest selection, colour extraction, assay-specific calculation, result storage and CSV export within a single user-guided workflow.

DiColorimetry includes three modules. The TPC module uses the red channel of the RGB model and a gallic acid calibration, while the RSA module uses the selected combined red–green response. The standalone colorimetric camera extracts RGB, HSV, CIELAB and CIELCh data without applying a predefined analytical equation, allowing users to develop or adapt additional image-based methods. This module has also been applied to colour characterization of plant-derived syrups using another Android smartphone Motorola Edge 40 Neo 5 G (Motorola Mobility, Beijing, China) [28].

The methodological contribution of the present work lies in integrating previously separate image acquisition, ROI-based colour extraction, calibration or reference handling, assay-specific calculation, result storage and data export within a single mobile workflow, rather than in introducing a new smartphone-imaging principle or modifying the established Folin–Ciocalteu and DPPH assays. The method is intended for researchers, students and laboratory users requiring an accessible smartphone-assisted readout for established colorimetric assays. This study describes the complete workflow and evaluates its analytical performance for TPC and RSA determination in 40 commercial vegetable oil samples by comparison with UV/VIS spectrophotometry under controlled illumination and fixed imaging conditions.

## Method details

### Materials and samples

Forty commercially available vegetable oil samples were used to evaluate the analytical performance and applicability of the DiColorimetry-assisted smartphone colorimetric method. The samples represented ten botanical categories, with four independent commercial products included in each category. The analyzed oils were sea buckthorn, sunflower, rice bran, hemp, corn, grape seed, linseed, rapeseed, olive and milk thistle oils. The sample set included cold-pressed, extracted and extra virgin oils originating from different commercial brands and countries, thereby providing a broad range of commercially relevant vegetable-oil matrices. Detailed information on the oil type, declared processing method, country of origin and sample identification code is provided in Supplementary Table S1. All samples were kept in their original commercial glass packaging until analysis to maintain sample integrity and reduce handling-related variability.

#### Reagents and colorimetric assays

Two established colorimetric assays were adapted for use with the DiColorimetry application: the Folin–Ciocalteu reaction for total phenolic content (TPC) determination, adapted from Fuentes et al. [29] and the 2,2-diphenyl-1-picrylhydrazyl (DPPH) assay for radical scavenging activity (RSA) assessment, adapted from Ahmed et al. [30]. For both assays, reaction mixtures were prepared under controlled laboratory conditions and then analyzed using both ultraviolet–visible (UV/VIS) spectrophotometry and DiColorimetry-assisted smartphone digital image analysis. The same final reaction solutions were used for both detection approaches; therefore, the sample-preparation and colorimetric-reaction steps were identical, while only signal acquisition and data processing differed.

#### Preparation of calibration standards for total phenolic content (TPC) determination

Gallic acid obtained from Sigma-Aldrich Chemie Ltd. (Steinheim, Germany) was used as the calibration standard for total phenolic content determination. A series of gallic acid standard solutions was prepared in 96% ethanol within the concentration range of 1–160 mg L⁻¹. The final calibration series consisted of nine concentration levels: 1, 2, 3, 5, 10, 20, 40, 80 and 160 mg L⁻¹. This calibration range was selected based on the preliminary linearity evaluation described in the Method validation section. For each calibration point, 0.5 mL of gallic acid standard solution was mixed with 2.5 mL of 0.2 M Folin-Ciocalteu phenol reagent (Sigma-Aldrich Chemie Ltd., Steinheim, Germany). After 5 min of reaction, 2.0 mL of 7.5% sodium carbonate solution (Sigma-Aldrich Chemie Ltd., Steinheim, Germany) was added. The mixtures were kept at room temperature (21 ± 1 °C) for 2 h to allow color development. After incubation, the colored solutions were transferred to 2.5 mL disposable polystyrene macro cuvettes (BrandTech™ Scientific, Inc., Essex, CT, USA). The absorbance of each standard was measured at 760 nm using an Agilent Cary 60 UV/VIS spectrophotometer (Agilent Technologies, Inc., Santa Clara, CA, USA). Digital images of the same calibration solutions were acquired under the standardized smartphone imaging conditions described below. A 96% ethanol solution was used as the reference solution for both UV/VIS spectrophotometric and smartphone-based measurements. Separate calibration curves were generated from the UV/VIS absorbance values and the DiColorimetry-derived image data. For smartphone-based TPC quantification, the mean intensity of the red channel (R) of the RGB color model was converted into an absorbance-like response relative to the reference intensity, *I_0_* using the equation provided in the calculation of smartphone-derived analytical signals subsection. Although the standalone colorimetric camera module supports additional color spaces, only the RGB red-channel response was used for the reported TPC quantification.

#### Determination of total phenolic content in vegetable oils

For total phenolic content determination, 0.5 g of vegetable oil sample was transferred into a 15 mL conical polypropylene tube (Sarstedt AG & Co. KG, Nümbrecht, Germany). Then, 2.5 mL of 0.2 M Folin-Ciocalteu phenol reagent (Sigma-Aldrich Chemie Ltd., Steinheim, Germany) was added and the mixture was vortexed for 1 min using an IKA Vortex 3 (IKA®-Werke GmbH & Co. KG, Staufen, Germany). After 5 min, 2.0 mL of 7.5% sodium carbonate solution (Sigma-Aldrich Chemie Ltd., Steinheim, Germany) was added and the mixture was vortexed for 30 s at speed 7. The reaction mixture was incubated in the dark for 2 h at room temperature (21 ± 1 °C). After incubation, the reaction mixture was divided into aliquots and transferred from the 15 mL conical tube into 2.0 mL polypropylene microcentrifuge tubes (Eppendorf™ Safe-Lock Tubes, Fisher Scientific UK Ltd., Loughborough, UK). The aliquots were centrifuged at 10.000 rpm for 5 min using a NRK5424 Microtube Centrifuge equipped with a BRK5424 fixed-angle rotor (Pro-Research, Centurion Scientific Ltd., Chichester, UK). Following centrifugation, the clarified coloured phase was transferred to 2.5 mL disposable polystyrene macro cuvettes (BrandTech™ Scientific, Inc., Essex, CT, USA) with dimensions of 12.5 × 12.5 × 45 mm.

The same reacted sample solution was analysed using both ultraviolet–visible (UV/VIS) spectrophotometry and DiColorimetry-assisted smartphone digital image analysis. The absorbance was measured at 760 nm using an Agilent Cary 60 UV/VIS spectrophotometer (Agilent Technologies, Inc., Santa Clara, CA, USA) against 96% ethanol as the reference solution. Digital images of the same sample solutions were acquired under the standardized illumination, camera-to-cuvette distance, cuvette-positioning and image-acquisition conditions described in the Equipment and smartphone imaging setup subsection. A cuvette containing 96% ethanol was used to obtain the smartphone reference intensity, *I_0_*.

For smartphone-based quantification, the mean red-channel intensity, *I_R_* within the predefined region of interest was converted into a digital absorbance-like value according to Eq. (1):

(1) $$A_{R}=-\log_{10}\left(\frac{I_{R}}{I_{0}}\right)$$

where, *A_R_* is the smartphone-derived red-channel absorbance-like response, *I_R_* is the mean red-channel intensity of the reacted sample solution and *I_0_* is the mean red-channel intensity of the 96% ethanol reference solution.

The red channel was selected for TPC quantification based on the channel-comparison results reported in our previous study [26]. In the present study, the red-channel response was recalibrated using the Samsung Galaxy A33 5 G smartphone under the specified image-acquisition conditions.

Separate gallic acid calibration curves were constructed for the UV/VIS and smartphone detection approaches. For UV/VIS measurements, the absorbance at 760 nm was used as the analytical response, whereas the smartphone calibration was based on *A_R_*. Total phenolic content was calculated separately for each detection approach using Eq. (2):

(2) $$TPC=\left(\;\frac{A-b)}{m}\right)\times \left(\frac{V}{m_{s}}\right)$$

where, *A* is the analytical response, *b* is the intercept of the corresponding calibration equation, *m* is its slope, *V* is the total reaction-solution volume in litres and *m_s_* is the vegetable-oil sample mass in kilograms. The results were expressed as milligrams of gallic acid equivalents per kilogram of oil (mg GAE kg⁻¹).

Each reacted sample solution was transferred to a single cuvette. The same cuvette was measured ten times by UV/VIS spectrophotometry and imaged ten times using the smartphone. The resulting measurements were used to assess repeatability of signal acquisition rather than repeatability of the complete sample-preparation procedure.

#### Determination of radical scavenging activity (RSA) using the DPPH assay

Radical scavenging activity was determined using the DPPH assay, adapted for both UV/VIS spectrophotometric measurement and smartphone-based image analysis. DPPH reagent (Sigma-Aldrich Chemie Ltd., Steinheim, Germany) was freshly prepared each day in 96% ethanol at a concentration of 0.02 g L⁻¹. The solution was stored at 4 °C in a light-protected volumetric flask in a refrigerator until use.

For each vegetable oil sample, 0.1 mL of oil was mixed with 3.0 mL of DPPH solution in a 15 mL conical polypropylene tube (Sarstedt AG & Co. KG, Nümbrecht, Germany). The mixture was vortexed for 30 s using an IKA Vortex 3 (IKA®-Werke GmbH & Co. KG, Staufen, Germany) at speed 7. The tubes were then incubated for 30 min in the dark at room temperature (21 ± 1 °C). After incubation, the mixtures were centrifuged at 3.000 rpm for 5 min in the same 15 mL conical polypropylene tubes using a K2015R refrigerated centrifuge equipped with a BRK5510 swing-out rotor (Pro-Research, Centurion Scientific Ltd., Chichester, UK). Following centrifugation, the clarified coloured solution was transferred to 2.5 mL disposable polystyrene macro cuvettes (BrandTech™ Scientific, Inc., Essex, CT, USA) with dimensions of 12.5 × 12.5 × 45 mm. A DPPH reference solution containing DPPH reagent without vegetable oil was subjected to the same 30 min incubation period. The absorbance of the DPPH reference solution and each reacted sample solution was measured at 517 nm using an Agilent Cary 60 UV/VIS spectrophotometer (Agilent Technologies, Inc., Santa Clara, CA, USA), with 96% ethanol used as the blank.

Digital images of the same DPPH reference and reacted sample solutions were acquired under the standardized illumination, camera-to-cuvette distance, cuvette-positioning, and image-acquisition conditions described in the Equipment and smartphone imaging setup subsection. A cuvette containing 96% ethanol was used to obtain the smartphone blank intensity, *I_0_*_._

For smartphone-based RSA determination, the mean red and green channel intensities were extracted from the predefined region of interest. The combined red–green signal was calculated according to Eq. (3):

(3) $$I_{RG}=\frac{I_{R}+\;I_{G}}{2}$$

where, *I_R_* and *I_G_* are the mean red- and green-channel intensities, respectively and *I_RG_* is the mean combined red–green intensity.

The *I_RG_* values of the DPPH reference and sample solutions were converted into digital absorbance-like responses according to Eq. (4):

(4) $$A_{RG}=-\log_{10}\left(\frac{I_{RG}}{I_{0,\;RG}}\right)$$

where, *A_RG_* is the smartphone-derived absorbance-like response, *I_RG_* is the combined red–green intensity of the DPPH reference or reacted sample solution and *I_0,RG_* is the corresponding combined red–green intensity of the 96% ethanol blank.

Radical scavenging activity was calculated separately for UV/VIS spectrophotometry and smartphone image analysis using Eq. (5):

(5) $$RSA\;\left(\%\right)=\frac{A_{DPPH}-A_{sample}\;}{A_{DPPH}}\;\times 100$$

where, *A_DPPH_* is the absorbance of the DPPH reference solution and *A_sample_* is the absorbance of the DPPH – vegetable oil reaction mixture after 30 min. For UV/VIS calculations, the absorbance measured at 517 nm was used, whereas for smartphone calculations, the corresponding *A_RG_* value was used.

The RG channel combination was selected for the reported RSA results based on the preliminary comparison of the individual R, G, and B channels and the combined RG, RB, GB and RGB responses. The RG response showed the closest agreement with the UV/VIS reference results across the investigated vegetable-oil samples, whereas signals containing the B channel showed substantially lower analytical agreement. The complete channel-comparison results are presented in Supplementary Table S2.

No chemical antioxidant standard was used for the quantitative expression of RSA. Therefore, the results were reported as percentage inhibition of the DPPH radical rather than as Trolox or other antioxidant equivalents.

#### Equipment and smartphone imaging setup

Reference measurements were performed using a Cary 60 UV/VIS spectrophotometer (Agilent Technologies, Inc., Santa Clara, CA, USA). Smartphone-based image acquisition was carried out using a Samsung Galaxy A33 5 G smartphone (Samsung Electronics Vietnam Thai Nguyen Co., Ltd., Van Xuan, Vietnam) and a Puluz portable photo studio light box (Puluz Technology Ltd., Shenzhen, China), which provided standardized illumination for image capture. The photo box was equipped with 40 LED lamps and was operated at a fixed colour temperature of 6500 K throughout all TPC and RSA image-acquisition experiments. The illumination setting was not changed between the acquisition of blanks, calibration standards, DPPH reference solutions and reacted sample solutions.

For image acquisition, the smartphone was positioned horizontally outside the front opening of the photo box, with the main rear camera facing the cuvette. A fixed camera-to-cuvette distance of 12 cm, defined as the distance between the smartphone camera lens and the centre of the cuvette, was maintained throughout image acquisition. Disposable 2.5 mL polystyrene macro cuvettes with dimensions of 12.5 × 12.5 × 45 mm were used for the measurements. The cuvettes were placed upright in a fixed central position, with the front optical surface perpendicular to the camera axis, against a uniform white background to improve contrast and reproducibility.

Depending on the assay, the cuvettes contained 96% ethanol as a blank, reacted gallic acid calibration solutions, the DPPH reference solution, or the clarified coloured reaction solution obtained from a vegetable oil sample. All standards, blanks, reference solutions and samples were placed under identical optical conditions during image capture to ensure consistency between measurements. The smartphone position, cuvette position, illumination, background and camera-to-cuvette distance remained unchanged within each assay-specific image-acquisition series.

**Fig. 1:**
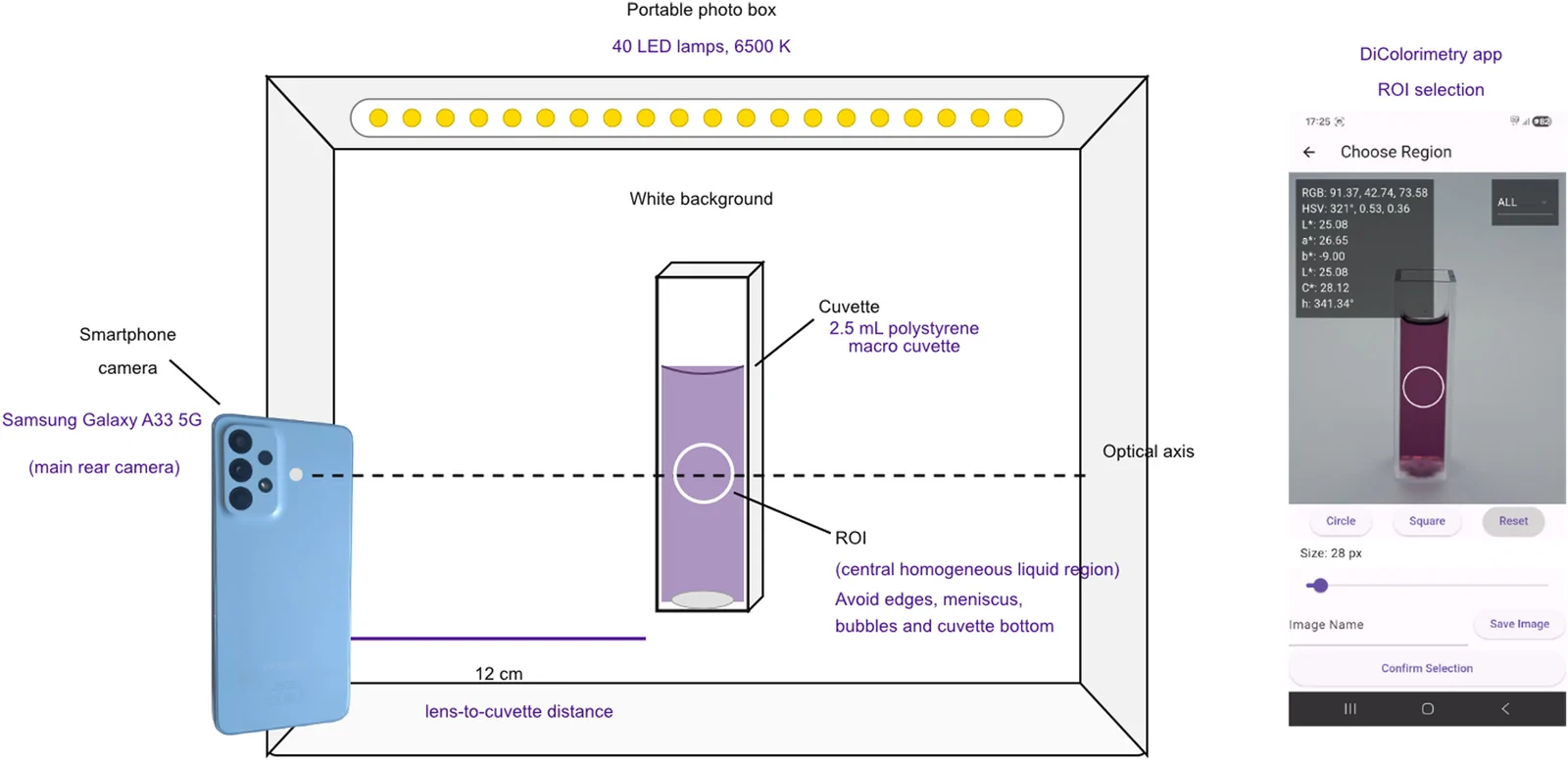
Standardized smartphone image-acquisition setup used for DiColorimetry analysis. The Samsung Galaxy A33 5 G smartphone was positioned outside the front opening of the portable photo box, with the main rear camera aligned perpendicular to the central region of the cuvette. The distance from the camera lens to the centre of the cuvette was fixed at 12 cm. Images were acquired under 40 LED lamps at 6500 K against a uniform white background. The ROI was selected within the homogeneous central liquid region, avoiding the meniscus, cuvette edges, bubbles, residual sediment and the cuvette bottom. The schematic is not drawn to scale.

Colour information was extracted from a manually selected region of interest located within the homogeneous central part of the liquid phase. The ROI was positioned below the meniscus and above the bottom of the cuvette to avoid the air–liquid interface, cuvette edges and any residual sediment. The same ROI shape and approximately the same position were used for blanks, calibration or reference solutions and samples. A schematic representation of the imaging setup is provided in Fig. 1.

Images were saved in JPG format with a 3:4 aspect ratio. A 1× main rear-camera setting was used, with autofocus, automatic white balance and automatic exposure control enabled. Exposure compensation was set to 0.0 EV Camera sensitivity was automatically adjusted according to the fixed illumination conditions within the photo box. Because automatic white balance and exposure were enabled, all assay-specific blank, calibration, reference, and sample images were acquired sequentially under unchanged illumination and imaging geometry. This procedure ensured internal comparability of the colour data within each measurement series.

#### DiColorimetry Android application

Colorimetric analysis was performed using DiColorimetry version 1.2, a custom Android application designed and developed by the authors for smartphone-assisted analytical colour measurements. The application was developed using the Flutter framework and the Dart programming language.

The analytical workflow combines two linked components: an established colorimetric assay that generates the measurable colour response and a DiColorimetry-based digital readout that performs image acquisition or input, ROI-based colour extraction, signal processing and assay-specific calculation. The methodological contribution of DiColorimetry lies in integrating these digital-analysis and calculation steps within a single mobile workflow rather than in introducing a new colorimetric reaction or a new principle of smartphone imaging.

The application includes three main functional modules: total phenolic content (TPC) determination, radical scavenging activity (RSA) assessment and a standalone colorimetric camera mode. The TPC and RSA modules provide assay-specific analytical calculations, whereas the standalone camera module enables general extraction of colour coordinates from digital images.

The analytical logic implemented in the TPC and RSA modules was based on the authors’ previously developed smartphone-image-analysis workflows [26,27]. In DiColorimetry, the previously separate image analysis, signal transformation, calibration and calculation operations were integrated into a single mobile workflow that does not require external scripts during routine use.

The application supports manual entry of colour values, real-time image acquisition using the smartphone camera and analysis of images stored on the device. Following image capture or upload, the user manually selects a circular or square region of interest (ROI) and the mean colour values of the pixels within the selected region are calculated.

The application displays analytical results and, where applicable, calibration curves, regression equations, and coefficients of determination. Colour data and analytical results can be exported in comma-separated values (CSV) format for documentation and further statistical analysis. Unlike general-purpose colour-picker applications, DiColorimetry integrates ROI-based colour extraction, assay-specific signal processing, calibration and final analytical calculation within one user-guided application. Compared with conventional UV/VIS workflows, the intended advantage of DiColorimetry is not superior metrological performance but the integration of analytical readout and calculation steps within a integrated mobile workflow. For TPC determination, the calibration model is generated directly within DiColorimetry from the smartphone responses obtained for the gallic acid standards; therefore, a UV/VIS-derived calibration curve is not required for routine smartphone analysis. For RSA determination, no concentration-based calibration curve is required because the result is calculated relative to the DPPH reference response. DiColorimetry therefore reduces dependence on separate image-processing, regression and calculation software while retaining the established colorimetric assay and calibration requirements where applicable. These practical advantages do not eliminate the need for controlled image acquisition, appropriate sample preparation, device-specific calibration, and matrix-specific analytical evaluation. Detailed operation of the three modules is described in the following workflow sections.

The DiColorimetry Android APK file (version 1.2), supporting documentation and software metadata are available through the Zenodo repository listed in the Resource availability and Supplementary material sections [31]. The application is distributed under a Custom Academic and Non-Commercial Licence.

#### Total phenolic content determination workflow

**Fig. 2:**
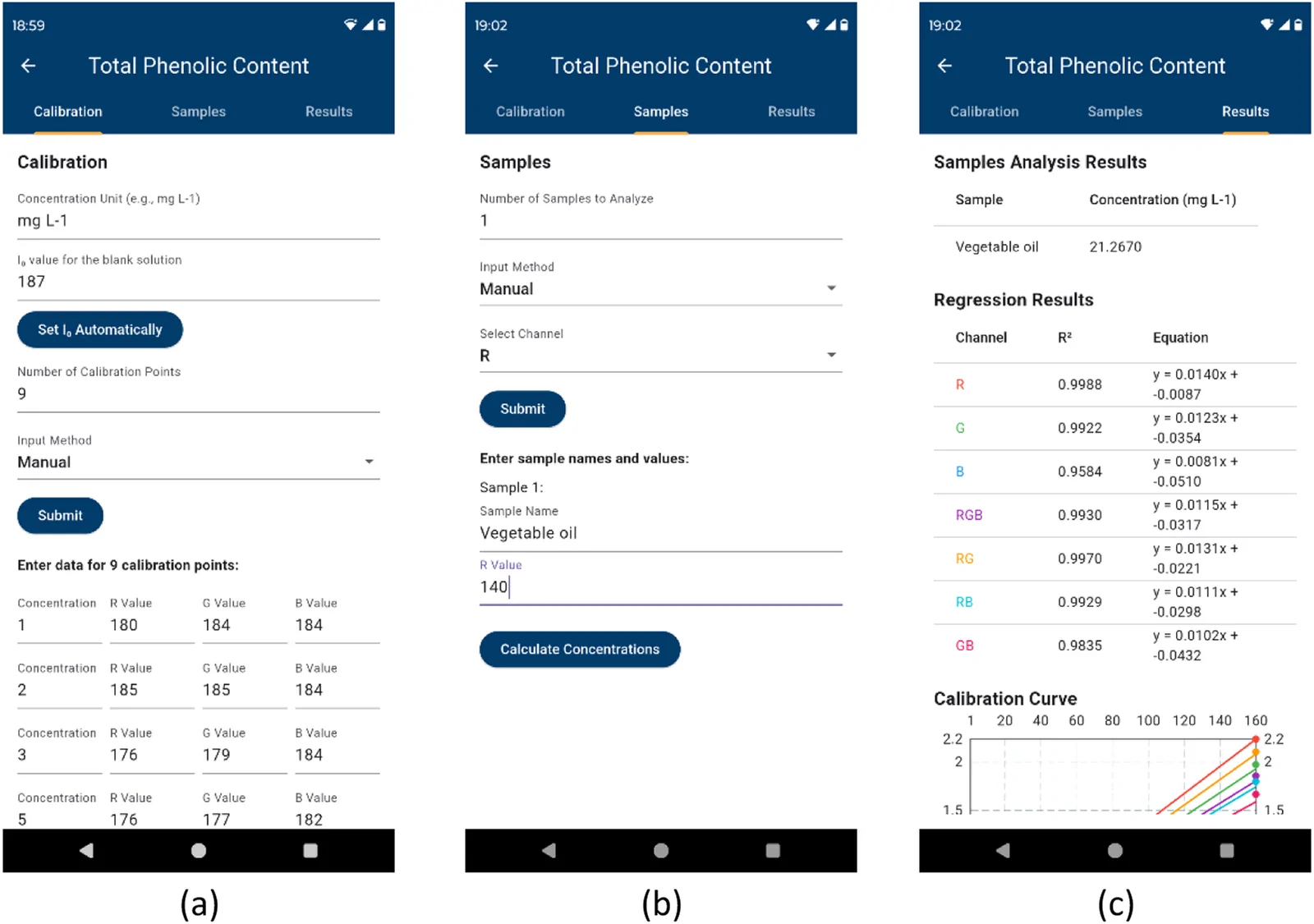
Operational stages of the total phenolic content analysis mode in the DiColorimetry application: (a) Entry or acquisition of blank and calibration data; (b) Entry or acquisition of sample colour values and calculation of concentration; and (c) Display of the calculated result, calibration equation, coefficient of determination, and calibration curve.

Fig. 2 illustrates the operational stages of the total phenolic content analysis mode in the DiColorimetry application. This module enables image-based colorimetric analysis by converting RGB color intensity values into absorbance-like responses and calibration-based quantitative analytical results.

Fig. 2**(a)** presents the calibration data input interface, where the user manually enters the intensity value of the blank solution (I₀), as well as the number of calibration points. Data can be entered manually using values derived from experimentally obtained images. Alternatively, the “Set I₀ Automatically” function enables automated acquisition of this value either in real time using the smartphone camera or from a previously saved image uploaded from the smartphone memory. Depending on the number of calibration points, the user enters the corresponding concentration for each point and either manually or automatically enters the respective R, G and B values. To proceed with the calculations, the entered data must be confirmed by pressing the “Submit Calibration Points” button.

Fig. 2**(b)** illustrates the sample input stage, in which the user specifies the object to be analyzed, for example, “vegetable oil”, along with its color intensity values. Based on the previously entered calibration data, the application allows the user to select an individual color channel, R, G, or B, or combined channel options, including RGB, RG, RB, or GB. Following either manual or automatic data entry, the application calculates the sample concentration using a calibration model based on linear regression. For the TPC results reported in the present study, the red channel (R) was selected as the analytical response based on its calibration performance and agreement with UV/VIS spectrophotometry.

Fig. 2**(c)** shows the final result screen. The application displays the calculated sample concentration, expressed in the units defined during calibration, together with the calibration equation, slope and intercept, coefficient of determination (R²) and calibration curves for each analyzed color channel or channel combination. The acquired data can be exported in CSV format for further statistical analysis, documentation, or storage on other devices.

The TPC module is designed for analyses requiring calibration-based concentration determination from digital images. In this workflow, RGB color channel values are converted into absorbance-like values according to the Beer–Lambert principle, after which calibration curves, regression equations, and corresponding R² coefficients are generated. The application performs the conversion, calibration modelling and concentration calculation within the same workflow, thereby avoiding the need to transfer RGB values to separate calculation software. Although the workflow was applied here to vegetable oil samples, the calibration structure can be adapted to other sample matrices only after establishing and validating an appropriate colorimetric reaction, calibration range and analytical channel for the intended application.

### Radical scavenging activity determination workflow

**Fig. 3:**
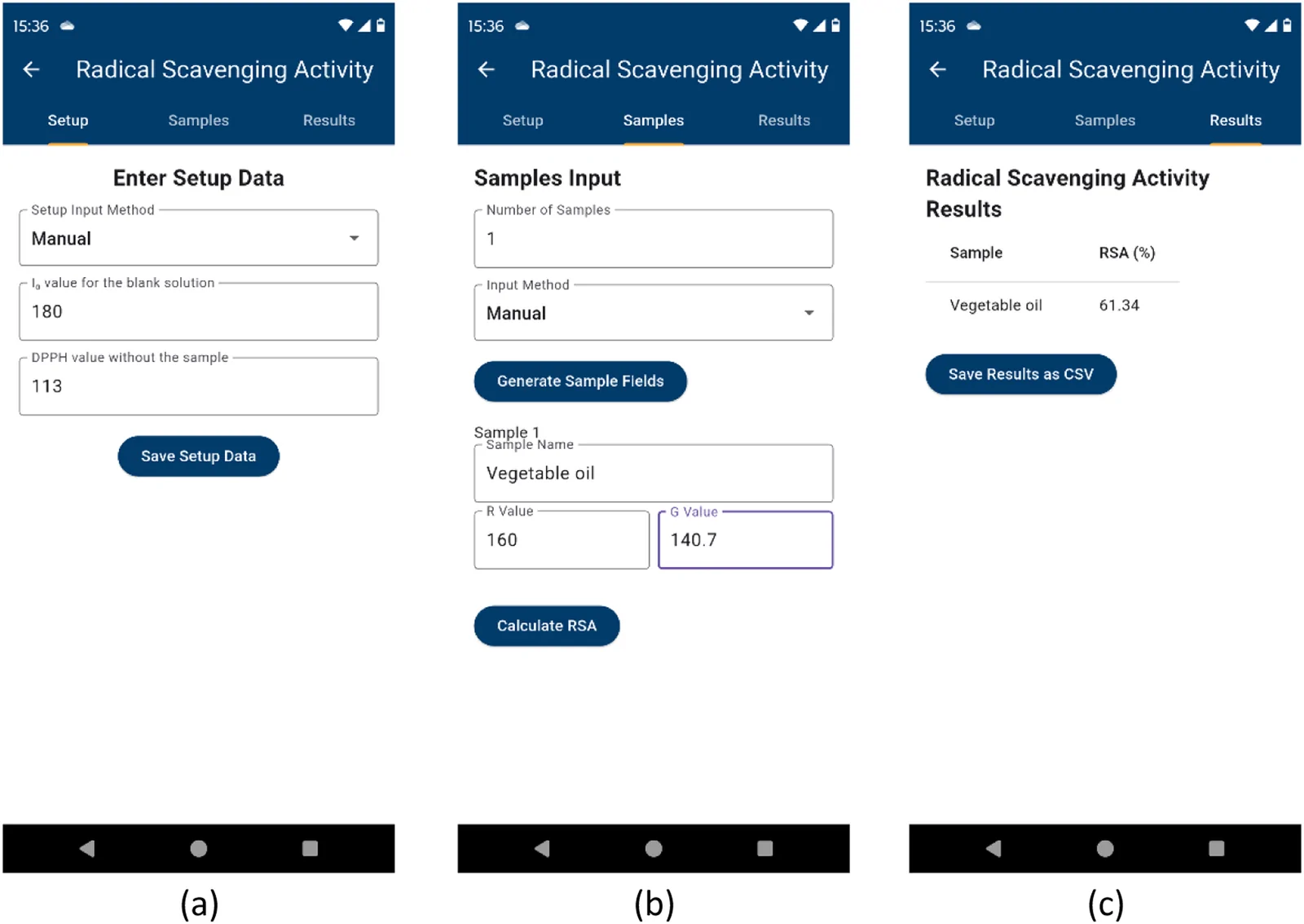
Operational stages of the radical scavenging activity analysis mode in the DiColorimetry application: (a) Entry or acquisition of the blank intensity (*I_0_*) and DPPH reference data; (b) Entry or acquisition of sample R and G channel values; and (c) Display and CSV export of the calculated RSA value.

Fig. 3 illustrates the operational stages of the radical scavenging activity analysis mode in the DiColorimetry application. This module is adapted to the DPPH assay and uses digitally acquired images and RGB color channel values to calculate the percentage of radical scavenging activity (RSA). Unlike the TPC module, the RSA module does not require a concentration-based calibration curve because the result is calculated from the relative decrease in the DPPH signal.

Fig. 3**(a)** presents the initial setup stage, where the user enters the color intensity value of the blank solution (I₀) and the DPPH reference value measured without the sample. These parameters are required for calculating the decrease in absorbance associated with DPPH radical reduction in the presence of antioxidant compounds. Data can be entered manually, acquired in real time using the smartphone camera, or uploaded from a previously saved image. To continue to the sample input section, the setup data must be saved by selecting the “Save Setup Data” button.

Fig. 3**(b)** illustrates the sample input stage. The user first specifies the number of samples, after which the application generates the required input fields. Three input modes are available: manual entry, real-time camera capture and image upload. For each sample, the user enters the sample name and the corresponding R and G color channel values. If real-time camera capture or image upload is selected, the application automatically extracts the R and G values and displays the mean RG value used in the RSA calculation. In manual mode, the mean RG value is calculated from the individually entered R and G channel data. The combined RG response was used for all RSA results reported in this study because it showed the closest agreement with the UV/VIS measurements at 517 nm.

Fig. 3**(c)** shows the final result screen, where the calculated radical scavenging activity is displayed as RSA. The result is expressed as percentage DPPH radical inhibition. The results can be exported in CSV format for further processing, statistical analysis, or documentation. This workflow enables smartphone-based interpretation of the DPPH assay under standardized image acquisition conditions without requiring external spreadsheet-based calculation of the final RSA value.

### Colorimetric camera workflow

**Fig. 4:**
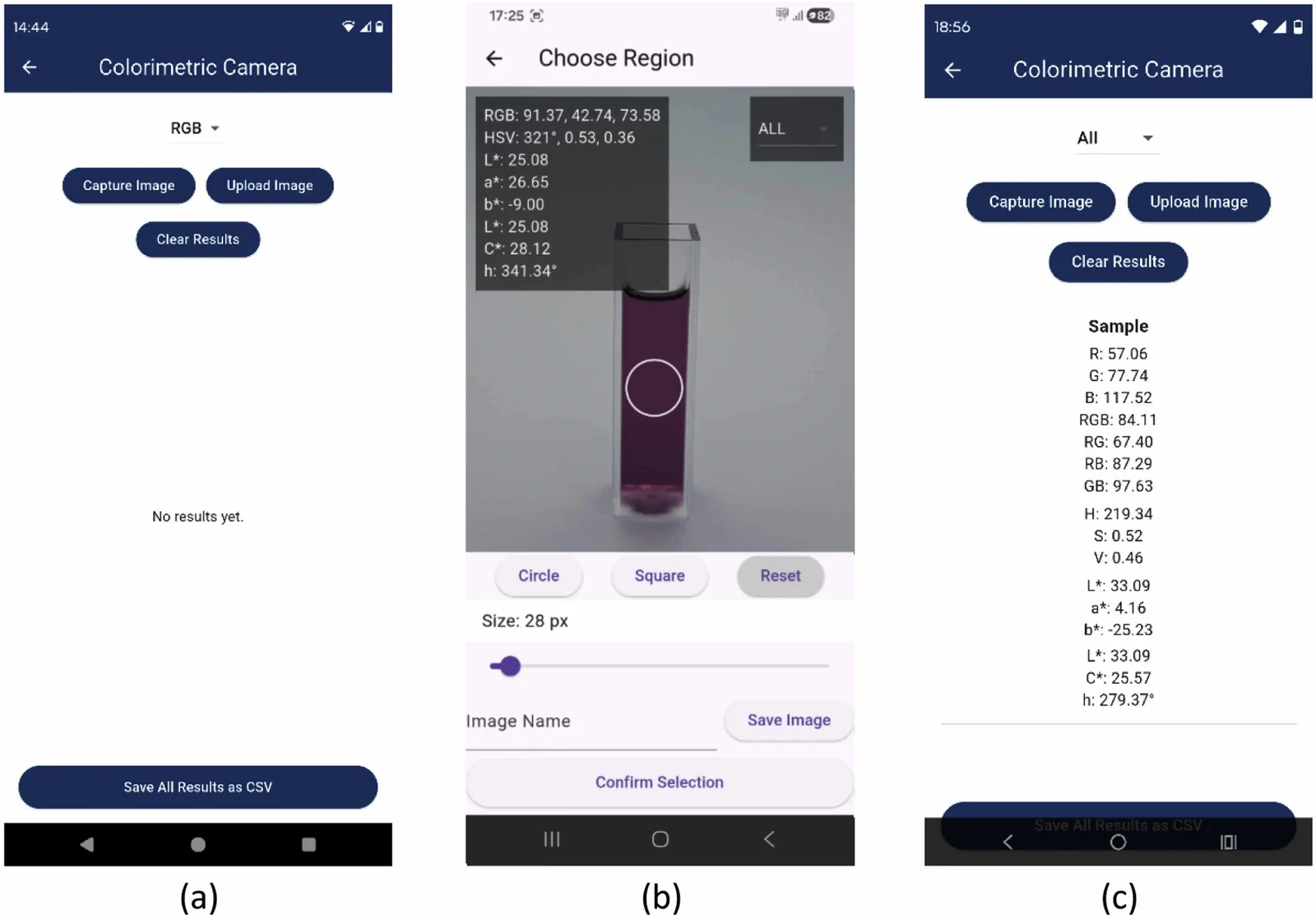
Operational stages of the standalone colorimetric camera mode in the DiColorimetry application: (a) Selection of real-time image acquisition or image upload; (b) Manual positioning and adjustment of a circular or square region of interest; and (c) Display and CSV export of colour parameters in the selected colour space.

Fig. 4 presents the operational stages of the standalone colorimetric camera mode in the DiColorimetry application. This mode enables image-based color characterization and can be used independently from the TPC and RSA analytical workflows. Unlike the assay-specific modules, the standalone camera mode reports colour coordinates but does not automatically calculate TPC, RSA, or another analyte concentration.

Fig. 4**(a)** shows the initial interface of the colorimetric camera mode. The user can either capture a new image in real time using the smartphone camera or upload an existing image from the device memory. Before analysis, the user selects the required color space from the available options. The application supports RGB, HSV, CIELAB, CIELCh and an “All” option for displaying the full set of available color parameters. The “All” option reports all supported parameters simultaneously but does not combine them into a single analytical result.

Fig. 4**(b)** illustrates the region of interest (ROI) selection step. The user manually selects the image area to be analyzed using either a circular or square region of interest. The size and position of the selected region can be adjusted to target the relevant part of the sample image. Before confirming the selection, the image can be enlarged and repositioned to improve the accuracy of ROI placement. If a new selection is required, the user can reset the selected region and repeat the procedure.

During ROI selection, the application displays color values in real time for the selected image area. After the region of interest is confirmed, the application calculates the corresponding color parameters according to the selected color space. For the standardized TPC and RSA measurements reported in this study, the ROI was positioned within the homogeneous central part of the liquid phase, below the meniscus and above the cuvette base, as described in the Equipment and smartphone imaging setup subsection.

Fig. 4**(c)** shows the result display screen. The acquired color data are presented in the selected coordinate system and the user can assign a name to the analysis. When the “All” option is selected, the application displays the complete set of supported color parameters, including RGB, HSV, CIELAB, CIELCh. The results can be exported in CSV format for further statistical processing, documentation, or integration into other data analysis environments. The user can also clear the results and start a new image acquisition or upload process.

The colorimetric camera mode expands the DiColorimetry application beyond TPC and RSA calculations by providing a general-purpose tool for digital color analysis of samples, solutions and other image-based colorimetric systems. However, any use of this mode for quantitative determination of another analyte requires the establishment of an appropriate reaction, analytical colour parameter, calibration model and independent validation. The HSV, CIELAB and CIELCh systems were available in the standalone camera mode but were not used for the TPC and RSA quantification reported in this study.

#### Analytical performance calculations and statistical analysis

The analytical performance of the DiColorimetry-assisted workflows was evaluated by comparison with reference ultraviolet–visible (UV–Vis) spectrophotometry. Method-comparison statistics were based on paired measurements of the same reacted and clarified solution in the same cuvette. The comparison therefore assessed the signal-acquisition, image-processing and calculation stages of the smartphone workflow, and not the recovery, matrix effects or repeatability of the complete sample-preparation and colorimetric-reaction procedure.

For TPC determination, separate linear calibration models were fitted by unweighted ordinary least-squares regression to the UV–Vis and DiColorimetry responses obtained for nine gallic acid standards covering the range 1–160 mg L^−1^, each prepared and measured in ten replicates. Calibration linearity was evaluated from the coefficient of determination (R^2^) and, in addition, from visual inspection of the residual plot to confirm the absence of systematic curvature over the calibration range.

The limit of detection (LOD) and the limit of quantification (LOQ) of the DiColorimetry TPC calibration were estimated from the standard deviation of the blank response and the slope of the calibration curve, according to Eqs. (6) and (7):

(6) $$\begin{array}{cccc} & LOD=\frac{3.3s_{0}}{b} & & \end{array}$$

(7) $$\begin{array}{cccc} & LOQ=\frac{10s_{0}}{b} & & \end{array}$$

where, s_0_ is the standard deviation of ten replicate red-channel absorbance-like responses (A_R_) obtained from independently prepared reagent blanks, and *b* is the slope of the DiColorimetry gallic acid calibration curve.

Signal-acquisition repeatability was evaluated using ten repeated UV/VIS readings and ten repeated smartphone images of the same prepared solution in the same cuvette (*n* = 10). Results were expressed as mean ± standard deviation and the relative standard deviation was calculated as RSD (%) = (SD / x̄) × 100. These measurements characterise instrumental-reading and image-acquisition repeatability only; they do not include the variability of independent sample preparations and must not be interpreted as method precision.

Method-comparison statistics were calculated from 40 paired vegetable-oil samples, each value being the mean of ten repeated signal acquisitions. For sample i, x_i_ denotes the UV–Vis result and y_i_ the corresponding DiColorimetry result. Ordinary least-squares regression was fitted as y = a + bx and 95% confidence intervals (CI) were calculated for the slope and the intercept. Agreement was assessed from whether the slope CI included 1 (absence of proportional bias) and the intercept CI included 0 (absence of constant bias). Because both methods are subject to measurement error, the least-squares model was used descriptively and was complemented by Bland–Altman analysis.

The paired difference was defined as d_i_ = y_i_ − x_i_. Mean bias was calculated as the arithmetic mean of the paired differences, and its 95% CI as d̄ ± t_0.975, n_ _−_ _1_ × SD_d_ / √n. The null hypothesis of zero mean bias was tested with a two-sided paired *t*-test. Mean absolute error (MAE) and root mean square error (RMSE) were calculated as MAE = n^−1^ Σ|d_i_| and RMSE = √(n^−1^ Σ d_i_^2^), with the summations taken over i = 1 to n and n = 40.

Mean relative bias, mean absolute relative deviation and maximum absolute relative deviation were calculated with the UV–Vis result as the denominator and expressed as percentages. Bland–Altman 95% limits of agreement were calculated as d̄ ± 1.96 SD_d_, where SD_d_ is the standard deviation of the paired differences. The normality of the differences was verified using the Shapiro–Wilk test, and the absence of proportional bias was checked by regressing the differences on the mean of each paired result.

Pearson correlation coefficients were used to describe the linear association between the paired results of the two detection approaches. The association between TPC and RSA was evaluated using both Pearson and Spearman correlation, the latter describing the monotonic association without assuming linearity. All statistical tests were two-sided and *p* < 0.05 was considered statistically significant. Results are reported to a number of significant figures consistent with the precision of the underlying measurements. All calculations were performed using Python 3.11.

### Method validation

The analytical performance of the DiColorimetry-assisted method was evaluated through method comparison with reference ultraviolet–visible (UV/VIS) spectrophotometry. For each assay, the same reacted and clarified solution was measured using both detection approaches, so the comparison specifically assessed the smartphone-based signal-acquisition, image-processing and calculation workflow rather than revalidation of the underlying Folin–Ciocalteu and DPPH chemical reactions.

The evaluation covered the two assay-specific quantitative modules of the application: total phenolic content (TPC) determination and radical scavenging activity (RSA) assessment. The evaluated parameters included calibration performance and sensitivity for TPC, agreement between DiColorimetry and UV/VIS results, and repeatability of repeated signal acquisition from the same prepared solution and cuvette. Because the repeated readings did not represent independent sample preparations, the reported precision parameters describe instrumental and image-acquisition repeatability rather than repeatability of the complete analytical procedure.

In addition, the standalone colorimetric camera module was functionally verified as a general-purpose tool for extracting RGB, HSV, CIELAB and CIELCh colour parameters from selected image regions. Because this module does not apply an assay-specific calibration or calculate a final analyte concentration, its assessment was limited to functional verification and was not considered quantitative analytical validation.

#### Analytical performance evaluation of total phenolic content determination

The total phenolic content determination workflow was evaluated by comparing paired TPC results obtained using DiColorimetry smartphone image analysis and reference UV/VIS spectrophotometry. Smartphone-based quantification was performed using the red channel of the RGB colour model. Calibration linearity, coefficient of determination, limit of detection and limit of quantification were evaluated using the gallic acid calibration series described above.

The comparison included all 40 commercial vegetable oil samples. The same reacted and clarified solution in the same cuvette was measured using both detection approaches. The results are presented in Table 1 as mean ± standard deviation of ten repeated signal acquisitions. These repetitions describe UV/VIS instrumental-reading repeatability and smartphone image-acquisition repeatability and do not represent independent sample preparations.

**Table 1:** Comparison of total phenolic content results obtained by UV/VIS spectrophotometry and DiColorimetry smartphone image analysis for 40 commercial vegetable oil samples.

Vegetable oils / Sample code		UV/VIS spectrophotometry	DiColorimetry red channel (R)	Vegetable oils		UV/VIS spectrophotometry	DiColorimetry red channel (R)
		TPC (mg GAE kg^−1^ oil)				TPC (mg GAE kg^−1^ oil)	
Olive	OL-ITA	179.2 ± 0.7	179.6 ± 1.1	Rapeseed	RA-LVA	89.5 ± 0.3	88.4 ± 0.5
	OL-ESP	280.6 ± 1.1	280.0 ± 1.7		RA-POL	85.9 ± 0.3	85.0 ± 0.5
	OL-PRT	400.1 ± 1.6	400.0 ± 2.5		RA-SWE	101.4 ± 0.4	100.7 ± 0.6
	OL-GRC	380.8 ± 1.5	380.2 ± 2.4		RA-DEU	80.7 ± 0.3	80.0 ± 0.5
Hemp	HE-LVA	181.4 ± 0.7	184.5 ± 1.1	Linseed	LI-ITA	35.7 ± 0.1	35.8 ± 0.2
	HE-LTU	160.7 ± 0.6	160.0 ± 1.0		LI-POL	28.6 ± 0.1	28.0 ± 0.2
	HE-POL	151.3 ± 0.6	150.4 ± 0.9		LI-DEU	76.1 ± 0.3	75.6 ± 0.5
	HE-DEU	145.7 ± 0.6	144.8 ± 0.9		LI-LVA	30.8 ± 0.1	30.4 ± 0.2
Sea buckthorn	SE-LVA	105.7 ± 0.4	104.6 ± 0.6	Sunflower	SU-DEU	30.7 ± 0.1	30.4 ± 0.2
	SE-EST	96.1 ± 0.4	95.0 ± 0.6		SU-POL	28.7 ± 0.1	28.0 ± 0.2
	SE-POL	91.2 ± 0.4	90.3 ± 0.6		SU-UKR	37.3 ± 0.1	36.8 ± 0.2
	SE-DEU	85.7 ± 0.3	85.2 ± 0.6		SU-ITA	29.3 ± 0.1	28.7 ± 0.2
Milk thistle	MI-LVA	90.5 ± 0.4	89.7 ± 0.6	Rice bran	RI-ITA	13.4 ± 0.1	12.9 ± 0.1
	MI-POL	80.6 ± 0.3	80.1 ± 0.5		RI-DEU	17.6 ± 0.1	17.0 ± 0.2
	MI-SVN	86.3 ± 0.3	85.4 ± 0.5		RI-IND	25.7 ± 0.1	25.2 ± 0.1
	MI-DEU	75.7 ± 0.3	75.0 ± 0.5		RI-USA	23.4 ± 0.1	22.8 ± 0.1
Corn	CO-ITA	13.2 ± 0.1	12.9 ± 0.1	Grape	GR-ITA	12.8 ± 0.1	12.4 ± 0.1
	CO—HUN	15.4 ± 0.1	15.0 ± 0.1		GR-ESP	15.3 ± 0.1	15.0 ± 0.1
	CO-POL	14.7 ± 0.1	14.3 ± 0.1		GR-FRA	10.8 ± 0.1	10.4 ± 0.1
	CO-UKR	16.7 ± 0.1	16.2 ± 0.1		GR-DEU	11.6 ± 0.1	11.2 ± 0.1

The TPC values obtained using the DiColorimetry application were closely comparable to those obtained by UV/VIS spectrophotometry across the analyzed vegetable oil samples. The paired results covered a broad TPC range, from approximately 10 to 400 mg GAE kg⁻¹, allowing the smartphone workflow to be assessed for samples with both low and high analytical responses.

In olive oil samples, the TPC values ranged from 179.6 ± 1.1 to 400.0 ± 2.5 mg GAE kg⁻¹ using smartphone image analysis, while UV/VIS spectrophotometry gave values from 179.2 ± 0.7 to 400.1 ± 1.6 mg GAE kg⁻¹. Similar agreement between the two methods was observed for hemp, sea buckthorn, milk thistle, rapeseed, linseed, sunflower, rice bran, corn and grape seed oils.

The lowest TPC values were observed in grape seed, corn and rice bran oils. In these samples, both methods produced comparable results, demonstrating that the smartphone-based workflow provided measurable responses at the lower end of the measured TPC range under the applied calibration and imaging conditions. Across all samples, the measured TPC values ranged from 10.8 to 400.1 mg GAE kg⁻¹ using UV/VIS spectrophotometry and from 10.4 to 400.0 mg GAE kg⁻¹ using DiColorimetry.

The observed differences among oil types may be associated with botanical origin, cultivar, and differences in the composition and distribution of phenolic compounds between the polar and lipid phases [[19], [20], [21]]. The higher TPC values observed in olive and hemp oils and the lower values in grape seed and rice bran oils were consistent with the findings of our previous study of commercial vegetable oils [26]. The low TPC values observed in corn seed oil and the variation among products within the same oil category may reflect differences in raw materials and commercial processing; however, these factors were not independently evaluated in the present study.

For DiColorimetry, linearity was evaluated over the gallic acid concentration range of 1 – 160 mg L⁻¹ using the red channel of the RGB colour model. The calibration equation obtained using the Samsung Galaxy A33 5 G smartphone was y = 0.0061x − 0.0022, with a coefficient of determination of R^2^ = 0.9989. The estimated LOD and LOQ were 1.008 and 3.055 mg L⁻¹, respectively, resulting in a quantitative range of 3.055 – 160 mg L⁻¹.

The corresponding analytical performance and method-comparison statistics are summarized in Table 2.

**Table 2:** Analytical performance characteristics and method-comparison statistics for total phenolic content determination.

Evaluation category	Parameter	UV/VIS spectrophotometry	DiColorimetry / method comparison
Analytical response	Wavelength or colour channel	760 nm	Red channel (R)
Calibration performance	Gallic acid calibration range (mg L⁻¹)	1 – 160	1 – 160
	Calibration equation^a^	y = 0.0115x + 0.0296	y = 0.0061x – 0.0022
	Coefficient of determination, (R²)	0.9997	0.9989
	Limit of detection, LOD (mg L⁻¹)	–	1.008
	Limit of quantification, LOQ (mg L⁻¹)	–	3.055
	Quantitative range (mg L⁻¹)	–	3.055– 160
Dataset	Number of paired vegetable-oil samples	40	40
	TPC result range (mg GAE kg⁻¹)	10.8 – 400.1	10.4 – 400.0
Signal-acquisition repeatability	Repeated readings or images per prepared solution	10	10
	Mean RSD (%)	0.460	0.662
	RSD range (%)	0.268 – 0.926	0.397 – 1.176
Method agreement	Linear regression equation^b^	-	y = 1.00101x – 0.56,196
	Regression slope	-	1.00,101
	Slope, 95% CI	-	0.99,872 – 1.00,330
	Regression intercept (mg GAE kg⁻¹)	-	−0.56,196
	Intercept, 95% CI (mg GAE kg⁻¹)	-	−0.84,965 to −0.27,427
	Pearson correlation coefficient, (*r*)	-	0.999,976
	Coefficient of determination, (R²)	-	0.999,951
	Mean bias, DiColorimetry − UV/VIS^c^ (mg GAE kg⁻¹)	-	−0.475
	95% CI of mean bias (mg GAE kg⁻¹)	-	−0.684 to −0.266
	SD of paired differences (mg GAE kg⁻¹)	-	0.654
	Mean absolute error, MAE (mg GAE kg⁻¹)	-	0.655
	Root mean square error, RMSE (mg GAE kg⁻¹)	-	0.802
	Mean relative bias (%)	-	−1.406
	Mean absolute relative deviation (%)	-	1.516
	Maximum absolute relative deviation (%)	-	3.731
	Bland–Altman 95% limits of agreement (mg GAE kg⁻¹)	-	−1.757 to 0.807
	Paired results with absolute relative deviation below 5%	-	40/40

Repeatability values were obtained from ten repeated signal acquisitions of the same prepared solution in the same cuvette. Method-comparison statistics were calculated from 40 paired samples.

ᵃ For the calibration equations, x is the gallic acid concentration (mg L⁻¹), while y is the corresponding analytical response: absorbance at 760 nm for UV/VIS spectrophotometry and the red-channel absorbance-like response for DiColorimetry.

ᵇ For the method-comparison regression, x is the UV/VIS TPC result and y is the DiColorimetry TPC result, both expressed as mg GAE kg⁻¹.

ᶜ Bias was calculated as DiColorimetry minus UV/VIS.

CI - confidence interval; LOD - limit of detection; LOQ - limit of quantification; RSD - relative standard deviation; MAE - mean absolute error; RMSE - root mean square error.

A very strong linear association was observed between the DiColorimetry and UV/VIS results, described by the regression equation y = 1.00101x − 0.56,196, where x represents the UV/VIS result and y represents the DiColorimetry result. The Pearson correlation coefficient was *r* = 0.999,976 and the coefficient of determination was R^2^ = 0.999,951. The regression slope was 1.00,101, with a 95% confidence interval of 0.99,872 – 1.00,330. Because this interval included 1, no substantial proportional bias was observed.

The mean bias, calculated as DiColorimetry minus UV/VIS, was −0.475 mg GAE kg⁻¹, with a 95% confidence interval of −0.684 to −0.266 mg GAE kg⁻¹. This small negative bias indicated that DiColorimetry generally produced slightly lower TPC results than UV/VIS spectrophotometry. The Bland–Altman 95% limits of agreement ranged from −1.757 to 0.807 mg GAE kg⁻¹, which was narrow relative to the overall measured TPC range.

The mean signal-acquisition RSD was 0.460% for UV/VIS spectrophotometry and 0.662% for DiColorimetry. The slightly higher RSD observed for smartphone image analysis may reflect additional variability associated with image acquisition and ROI positioning. Nevertheless, the mean RSD remained below 1% for both detection approaches. The individual RSD values ranged from 0.268% to 0.926% for UV/VIS and from 0.397% to 1.176% for DiColorimetry. These RSD values describe repeated signal acquisition from the same prepared solution and should not be interpreted as precision of the complete sample-preparation procedure.

The mean absolute relative deviation between the DiColorimetry and UV/VIS results was 1.516%, and the maximum observed absolute relative deviation was 3.731%. All 40 paired results showed absolute relative deviations below 5%. The mean absolute error was 0.655 mg GAE kg⁻¹ and the root mean square error was 0.802 mg GAE kg⁻¹. Overall, the results demonstrated close agreement between the two detection approaches across the investigated TPC range, with a small method-dependent negative bias. Accordingly, DiColorimetry may be used as a smartphone-based screening and quantitative readout approach under the specified calibration and image-acquisition conditions.

Compared with previously reported smartphone- and digital-image-based TPC approaches [11,13,[15], [16], [17], [18]], the principal practical contribution of DiColorimetry is the integration of ROI-based colour extraction, reference-signal handling, calibration, regression, final concentration calculation, and CSV export within a single application. Direct numerical comparison of analytical performance among studies is limited by differences in sample matrices, colour reactions, smartphone or imaging devices, image-acquisition conditions, calibration ranges, and reported validation parameters. Thus, the present workflow streamlines analytical handling and reduces dependence on separate image-processing or calculation tools, but it does not eliminate the need for assay-, matrix-, and device-specific calibration and analytical evaluation.

#### Analytical performance evaluation of radical scavenging activity determination

The analytical performance of the radical scavenging activity (RSA) workflow was evaluated by comparing paired results obtained using DiColorimetry smartphone image analysis and reference UV/VIS spectrophotometry for all 40 vegetable oil samples. The same reacted and clarified solution in the same cuvette was measured using both detection approaches. The reported mean values and standard deviations were calculated from ten repeated signal acquisitions and therefore describe UV/VIS instrumental-reading repeatability and smartphone image-acquisition repeatability rather than repeatability of the complete sample-preparation procedure.

The individual R, G, and B colour channels and the combined RG, RB, GB and RGB responses were initially evaluated to identify the most suitable analytical response for smartphone-based RSA calculation. The complete sample-level results obtained using all individual and combined colour responses are provided in Supplementary Table S2.

Among the evaluated responses, RG showed the closest agreement with UV/VIS spectrophotometry. The RG response produced a Pearson correlation coefficient of *r* = 0.996,781, a coefficient of determination of R^2^ = 0.993,573, a mean absolute error of 0.5575 percentage points and a mean absolute relative deviation of 0.7389%. In comparison, the individual R and G responses showed larger systematic deviations, whereas the B, RB, GB and RGB responses showed substantially higher errors relative to the UV/VIS results. The combined RG response was therefore selected for all final DiColorimetry RSA results reported in this study.

The paired RSA results obtained by UV/VIS spectrophotometry and DiColorimetry using the selected RG response for all 40 vegetable oil samples are presented in Table 3.

**Table 3:** Comparison of radical scavenging activity results obtained by UV/VIS spectrophotometry and DiColorimetry smartphone image analysis using the selected RG colour response for 40 commercial vegetable oil samples.

Vegetable oils Sample code		UV/VIS spectrophotometry RSA (%)	DiColorimetry, red–green channel (RG) RSA (%)
Sea buckthorn	SE-LVA	93.5 ± 0.3	93.2 ± 0.7
	SE-EST	85.0 ± 0.3	85.1 ± 0.7
	SE-POL	80.8 ± 0.2	80.6 ± 0.7
	SE-DEU	76.2 ± 0.2	75.7 ± 0.7
Sunflower	SU-DEU	80.7 ± 0.2	80.7 ± 0.6
	SU-POL	74.3 ± 0.2	73.8 ± 0.6
	SU-UKR	85.0 ± 0.3	85.8 ± 0.6
	SU-ITA	77.1 ± 0.2	77.3 ± 0.6
Rice bran	RI-ITA	67.6 ± 0.2	67.1 ± 0.6
	RI-DEU	73.0 ± 0.2	72.8 ± 0.6
	RI-IND	77.0 ± 0.2	77.3 ± 0.6
	RI-USA	75.2 ± 0.2	74.8 ± 0.6
Hemp	HE-LVA	95.7 ± 0.3	95.5 ± 0.7
	HE-LTU	89.4 ± 0.3	89.5 ± 0.7
	HE-POL	84.1 ± 0.2	84.0 ± 0.7
	HE-DEU	81.2 ± 0.2	81.0 ± 0.7
Corn	CO-ITA	61.5 ± 0.2	61.3 ± 0.6
	CO—HUN	71.6 ± 0.2	71.3 ± 0.6
	CO-POL	68.2 ± 0.2	67.7 ± 0.6
	CO-UKR	77.3 ± 0.2	76.7 ± 0.6
Grape	GR-ITA	73.3 ± 0.2	72.9 ± 0.6
	GR-ESP	88.7 ± 0.3	85.7 ± 0.6
	GR-FRA	59.1 ± 0.2	56.7 ± 0.6
	GR-DEU	66.2 ± 0.2	63.3 ± 0.6
Linseed	LI-ITA	68.3 ± 0.2	68.5 ± 0.6
	LI-POL	66.5 ± 0.2	66.8 ± 0.6
	LI-DEU	64.0 ± 0.2	63.6 ± 0.6
	LI-LVA	72.0 ± 0.2	72.5 ± 0.6
Rapeseed	RA-LVA	91.4 ± 0.3	91.7 ± 0.7
	RA-POL	87.6 ± 0.3	87.5 ± 0.7
	RA-SWE	95.2 ± 0.3	94.9 ± 0.7
	RA-DEU	81.0 ± 0.3	80.7 ± 0.7
Olive	OL-ITA	95.7 ± 0.3	95.5 ± 0.7
	OL-ESP	96.0 ± 0.3	96.3 ± 0.7
	OL-PRT	97.5 ± 0.3	97.2 ± 0.7
	OL-GRC	96.8 ± 0.3	96.4 ± 0.7
Milk thistle	MI-LVA	94.3 ± 0.3	94.6 ± 0.6
	MI-POL	84.2 ± 0.3	84.7 ± 0.6
	MI-SVN	89.5 ± 0.3	89.9 ± 0.6
	MI-DEU	78.7 ± 0.2	81.3 ± 0.6

Values are expressed as mean ± standard deviation of ten repeated signal acquisitions from the same prepared solution in the same cuvette (*n* = 10). These repetitions represent UV/VIS instrumental-reading repeatability and smartphone image-acquisition repeatability and do not represent independent sample preparations. RG denotes the combined red–green response selected for the final DiColorimetry RSA calculation.

The RSA values obtained using the DiColorimetry application were generally comparable to those obtained by UV/VIS spectrophotometry across the analyzed vegetable oil samples. Across all samples, RSA ranged from 59.1% to 97.5% using UV/VIS spectrophotometry and from 56.7% to 97.2% using the selected DiColorimetry RG response.

The highest radical scavenging activity was observed in olive oil samples. In this group, UV/VIS spectrophotometry gave values ranging from 95.7 ± 0.3% to 97.5 ± 0.3%, while DiColorimetry RG analysis gave values from 95.5 ± 0.7% to 97.2 ± 0.7%.

Hemp and rapeseed oils also showed high RSA values and close agreement between the two methods. For hemp oil, UV/VIS results ranged from 81.2 ± 0.2% to 95.7 ± 0.3%, while DiColorimetry RG results ranged from 81.0 ± 0.7% to 95.5 ± 0.7%. For rapeseed oil, UV/VIS results ranged from 81.0 ± 0.3% to 95.2 ± 0.3%, while DiColorimetry RG results ranged from 80.7 ± 0.7% to 94.9 ± 0.7%.

Sea buckthorn oil showed a broader RSA range, with UV/VIS values from 76.2 ± 0.2% to 93.5 ± 0.3% and DiColorimetry RG values from 75.7 ± 0.7% to 93.2 ± 0.7%. Lower RSA values were observed in grape seed oil, where UV/VIS results ranged from 59.1 ± 0.2% to 88.7 ± 0.3%, while DiColorimetry RG analysis produced values from 56.7 ± 0.6% to 85.7 ± 0.6%. These results indicate that the RSA workflow reproduced the general trend of the UV/VIS reference method across oils with both high and lower radical scavenging activity.

Differences in RSA among the investigated oil types may be associated with botanical origin, cultivar, processing and refining conditions, storage history and differences in both phenolic and non-phenolic antioxidant constituents. The comparatively high RSA values observed in olive, hemp and rapeseed oils were consistent with the results of our previous DPPH study of commercial vegetable oils [27]. The variation observed among products within the same oil category may reflect differences in raw materials and commercial processing; however, these factors were not independently evaluated in the present study.

Signal-acquisition repeatability and method-agreement statistics for the selected DiColorimetry RG response and UV/VIS spectrophotometry are summarized in Table 4.

**Table 4:** Analytical performance characteristics and method-comparison statistics for radical scavenging activity determination.

Evaluation category	Parameter	UV/VIS spectrophotometry	DiColorimetry / method comparison
Analytical response	Wavelength or colour channel	517 nm	Combined red–green channel (RG)
Dataset	Number of paired vegetable-oil samples	40	40
	RSA result range (%)	59.1 – 97.5	56.7 – 97.2
Signal-acquisition repeatability	Repeated readings or images per prepared solution	10	10
	Mean RSD (%)	0.300	0.807
	RSD range (%)	0.238 – 0.370	0.634 – 1.058
Method agreement	Linear regression equation^a^	-	y = 1.01841x − 1.69,472
	Regression slope	-	1.01,841
	Slope, 95% CI	-	0.99,151 – 1.04,531
	Regression intercept (percentage points)	-	−1.69,472
	Intercept, 95% CI (percentage points)	-	−3.87,962 to 0.49,018
	Pearson correlation coefficient, (*r*)	-	0.996,781
	Coefficient of determination, (R²)	-	0.993,573
	Mean bias, DiColorimetry − UV/VIS^b^ (percentage points)	-	−0.2125
	95% CI of mean bias (percentage points)	-	−0.5053 to 0.0803
	SD of paired differences (percentage points)	-	0.9154
	Mean absolute error, MAE (percentage points)	-	0.5575
	Root mean square error, RMSE (percentage points)	-	0.9286
	Mean relative bias (%)	-	−0.3108
	Mean absolute relative deviation (%)	-	0.7389
	Maximum absolute relative deviation (%)	-	4.3807
	Bland–Altman 95% limits of agreement (percentage points)	-	−2.0068 to 1.5818
	Paired results with absolute relative deviation below 5%	-	40/40

Repeatability values were obtained from ten repeated signal acquisitions of the same prepared solution in the same cuvette. Method-comparison statistics were calculated from 40 paired samples.

ᵃ For the method-comparison regression, x is the UV/VIS RSA result and y is the DiColorimetry RG RSA result, both expressed as %.

ᵇ Bias was calculated as DiColorimetry RG minus UV/VIS.

CI - confidence interval; RSD - relative standard deviation; MAE - mean absolute error; RMSE - root mean square error; RSA - radical scavenging activity; RG - combined red–green response.

The regression equation was y = 1.01841x − 1.69,472, with a Pearson correlation coefficient of *r* = 0.996,781 and a coefficient of determination of R^2^ = 0.993,573. The regression slope was 1.01,841 and its 95% confidence interval of 0.99,151–1.04,531 included the theoretical value of 1, indicating no substantial proportional bias. The 95% confidence interval of the intercept ranged from −3.87,962 to 0.49,018 percentage points and included zero.

The mean bias, calculated as DiColorimetry RG minus UV/VIS RSA, was −0.2125 percentage points, with a 95% confidence interval of −0.5053 to 0.0803 percentage points. The Bland–Altman 95% limits of agreement ranged from −2.0068 to 1.5818 percentage points. The confidence interval of the mean bias included zero, indicating that no substantial systematic difference was detected between the selected RG response and UV/VIS spectrophotometry.

The mean signal-acquisition RSD was 0.300% for UV/VIS spectrophotometry and 0.807% for DiColorimetry. The slightly higher variability observed for smartphone image analysis may be associated with additional variation introduced during image acquisition and ROI positioning. Nevertheless, the mean RSD remained below 1% for both detection approaches. Individual RSD values ranged from 0.238% to 0.370% for UV/VIS spectrophotometry and from 0.634% to 1.058% for DiColorimetry. These values describe repeated signal acquisition from the same prepared solution and should not be interpreted as precision of the complete sample-preparation procedure.

The mean absolute relative deviation between the DiColorimetry RG and UV/VIS results was 0.7389% and the maximum observed absolute relative deviation was 4.3807%. All 40 paired results showed absolute relative deviations below 5%. The mean absolute error was 0.5575 percentage points and the root mean square error was 0.9286 percentage points.

Overall, the selected DiColorimetry RG workflow showed close agreement with UV/VIS spectrophotometry across the investigated RSA range. These findings support its use as an assay-specific smartphone readout for DPPH radical-scavenging measurements under the specified conditions of controlled illumination, fixed imaging geometry, standardized ROI positioning and standardized sample preparation.

The principal contribution of the RSA module is the integrated handling of the blank, DPPH reference, sample RG response, and final percentage-inhibition calculation within the same application. Direct comparison with other smartphone-based antioxidant assays is restricted by differences in radical systems, matrices, reaction formats, analytical channels, and result-expression units [[12], [13], [14],^27^]. The workflow therefore provides an integrated assay-specific readout but remains dependent on controlled image acquisition and matrix-specific evaluation.

#### Correlation between total phenolic content and radical scavenging activity

A positive correlation was observed between total phenolic content (TPC) and radical scavenging activity (RSA) across the 40 vegetable oil samples. For the UV/VIS results, Pearson correlation analysis gave *r* = 0.7018 (*p* < 0.001), while the corresponding DiColorimetry results gave *r* = 0.6990 (*p* < 0.001). Spearman rank correlation coefficients were *ρ* = 0.8308 for UV/VIS and *ρ* = 0.8333 for DiColorimetry (*p* < 0.001 in both cases). These results indicate that oils with higher TPC generally exhibited higher RSA. However, TPC did not fully explain the variation in RSA, which may reflect differences in the composition and radical-scavenging activity of individual phenolic compounds, as well as contributions from non-phenolic antioxidants such as tocopherols and tocotrienols [32,33].

#### Functional verification and limitations of the standalone colorimetric camera module

The standalone colorimetric camera module was functionally verified as part of the DiColorimetry workflow. This module uses the same image acquisition and region-of-interest selection principles as the TPC and RSA modules, but it is provided as an independent tool for extracting colorimetric data without applying a predefined analytical formula.

The module allows the user to capture or upload an image, select a defined region of interest and extract color parameters in multiple color spaces and indices, including RGB, HSV, CIELAB and CIELCh. In the present application, the colorimetric camera mode is intended to support researchers in developing or adapting additional image-based colorimetric methods where accurate extraction of color parameters is required. Functional verification included image capture, image upload, manual ROI positioning, real-time display of colour values, calculation of the selected colour coordinates, saving of results and export in CSV format.

In the present study, functional verification and the analytical evaluation of the TPC and RSA modules were performed using a Samsung Galaxy A33 5 G smartphone. The standalone colour-extraction workflow has also been applied in a separate study of plant-derived syrups using a Motorola Edge 40 Neo 5 G (Motorola Mobility, Beijing, China) smartphone and DiColorimetry version 1.2. In that study, RGB and CIELAB coordinates were extracted from selected homogeneous regions of cuvette images acquired under standardized light-box conditions [28]. This application demonstrates the operational use of the standalone module with another Android smartphone and a different food matrix.

Unlike the TPC and RSA modules, the colorimetric camera module does not provide a final analyte concentration. Therefore, its use for other analytical purposes requires the user to establish an appropriate calibration model and perform separate validation for the intended analyte, matrix and colorimetric reaction. The module was not evaluated as a universal replacement for a calibrated colorimeter and no assay-independent claims are made regarding trueness, precision, limits of detection, limits of quantification, or inter-device reproducibility.

#### Limitations

The DiColorimetry-assisted method requires standardized image acquisition conditions. Variations in illumination, background color, camera-to-sample distance, cuvette position, image focus, or white balance may affect the extracted color values and therefore the calculated analytical results.

Although a portable light box, fixed imaging geometry, a white background and a defined region of interest were used to reduce image-acquisition variability, the smartphone camera operated with autofocus, automatic exposure and automatic white balance enabled. Consequently, residual variation associated with automatic camera processing cannot be completely excluded. Manual ROI positioning may also introduce additional operator-dependent variability, particularly when the analyzed area is not visually homogeneous.

Strong intrinsic sample colour, turbidity, emulsification, suspended particles, phase separation, or non-homogeneous colour development may interfere with ROI-based colour extraction and may limit the applicability of the workflow to certain matrices.

The analytical performance of the TPC and RSA workflows reported in the present study was evaluated using a Samsung Galaxy A33 5 G smartphone. DiColorimetry version 1.2 was also used with a Motorola Edge 40 Neo 5 G smartphone in a separate study of plant-derived syrups [28]. This separate application supports functional operation of the standalone colour-extraction workflow on another Android device, but it does not establish analytical equivalence between the Samsung and Motorola smartphones.

Earlier smartphone-based TPC and RSA studies used a Huawei P30 Lite smartphone [26,27]. However, those studies employed separate image-analysis tools rather than the current DiColorimetry version 1.2 application. They therefore support the underlying smartphone-based measurement concepts but should not be interpreted as functional or analytical verification of the current application on the Huawei device.

No controlled cross-device comparison was performed using the same samples, calibration standards, imaging conditions and analytical workflow. Differences in camera sensors, optics, internal image-processing algorithms, exposure control, white balance and colour rendering may affect the extracted colour values. Consequently, quantitative results obtained using different smartphone models should not be considered directly interchangeable without device-specific calibration or cross-device verification.

For method comparison, the same prepared and clarified solution in the same cuvette was measured by UV/VIS spectrophotometry and smartphone image analysis. Accordingly, the reported agreement primarily evaluates the detection and digital-readout stages of the workflows. It does not constitute an independent assessment of recovery, matrix effects, or repeatability of the complete sample-preparation procedure. Spike-recovery and matrix-effect experiments were not performed; therefore, the present evaluation is limited to comparison of the detection and digital-readout stages and does not constitute full validation of the complete analytical procedure.

The reported LOD and LOQ values apply to the smartphone-based TPC calibration under the specified assay and imaging conditions and should not be interpreted as universal, device-independent performance characteristics. The RSA module calculates percentage DPPH inhibition relative to the reference response and was not calibrated using an external antioxidant standard; therefore, assay-specific LOD and LOQ values were not established for RSA.

The standalone colorimetric camera module extracts color coordinates but does not provide a predefined analyte concentration or represent a calibrated instrumental colorimeter. Application to other analytes, reactions, matrices, smartphone models, or imaging environments requires the establishment of an appropriate calibration model and separate evaluation of linearity, precision, trueness, recovery, matrix effects, detection capability and robustness.

No formal usability or human-factors study involving non-specialist users was conducted; therefore, the present evaluation demonstrates the functional operation of the application rather than validated ease of use by untrained users.

Therefore, DiColorimetry should be regarded as an assay-specific smartphone readout and screening tool under controlled acquisition conditions rather than as a universally transferable analytical platform.

## Conclusions

DiColorimetry version 1.2 integrates ROI-based colour extraction, assay-specific signal transformation, calibration, and final analytical calculation for TPC and RSA within a single Android application. Using 40 commercial vegetable-oil samples, the red-channel TPC workflow and the combined RG RSA workflow showed close agreement with UV/VIS spectrophotometry. Mean signal-acquisition RSD values were below 1% for both detection approaches, and all paired TPC and RSA results showed absolute relative deviations below 5%. For smartphone-based TPC determination, the gallic acid calibration was linear from 1 to 160 mg L⁻¹, with LOD and LOQ values of 1.008 and 3.055 mg L⁻¹, respectively. The application also provides standalone extraction of RGB, HSV, CIELAB, and CIELCh coordinates and CSV export. Its principal practical advantage is the consolidation of assay-specific digital readout, calculation and data export within one mobile workflow, reducing dependence on separate image-processing and calculation tools rather than replacing the established chemical assay or reference instrumentation. However, the reported performance is assay-, device-, and imaging-condition-specific. Recovery, matrix effects, complete procedure repeatability, formal usability, and controlled inter-device equivalence were not established. DiColorimetry should therefore be used as a controlled smartphone-based screening and quantitative readout tool rather than as a universal replacement for calibrated analytical instrumentation.

## Ethics statements

This work did not involve human subjects, animal experiments, or data collected from social media platforms. Therefore, no ethics approval or informed consent was required.

## CRediT author statement

**Sanita Vucane**: Conceptualization, Methodology, Investigation, Formal analysis, Data curation. **Ingmars Cinkmanis**: Supervision, Conceptualization, Project administration, Software, Methodology. **Martins Sabovics:** Formal analysis, Writing - Original Draft, Writing - Review & Editing. **Heinrihs Cinkmanis:** Data curation, Software, Validation, Visualization.

## Declaration of interests

The authors declare that they have no known competing financial interests or personal relationships that could have appeared to influence the work reported in this paper.

## Data Availability

The DiColorimetry Android APK file and metadata are openly available via Zenodo:https://doi.org/10.5281/zenodo.17819093

## Appendix Supplementary materials

Image, application 1
