# Supplementary material for: DiColorimetry: A smartphone application for colorimetric analysis of phenolics and antioxidant activity in vegetable oils
Source: MethodsX. 2026 Sep 4;17:104144. doi: 10.1016/j.mex.2026.104144 (PMC13578464; doi:10.1016/j.mex.2026.104144)
Supplement: Supplementary file 1 [file mmc1.docx]

**Supplementary material *and/or* additional information [OPTIONAL]**

The supplementary material includes Tables S1 and S2. The DiColorimetry Android APK file, supporting documentation, and additional information required to reproduce the smartphone-assisted colorimetric workflow are available via Zenodo: <https://doi.org/10.5281/zenodo.17819093> [31].

**Supplementary Table S1.**

Characterisation of the commercially available vegetable oil samples used in the study

| Sample code | Vegetable oils | Processing method | Country |
| --- | --- | --- | --- |
| SE–LVA | Sea buckthorn | Cold pressed | Latvia |
| SE–EST |  |  | Estonia |
| SE–POL |  |  | Poland |
| SE–DEU |  |  | Germany |
| HE–LVA | Hemp | Cold pressed | Latvia |
| HE–LTU |  |  | Lithuania |
| HE–POL |  |  | Poland |
| HE–DEU |  |  | Germany |
| CO–ITA | Corn seed | Extracted | Italy |
| CO–HUN |  |  | Hungary |
| CO–POL |  |  | Poland |
| CO–UKR |  |  | Ukraine |
| GR–ITA | Grape seed | Extracted | Italy |
| GR–ESP |  |  | Spain |
| GR–FRA |  |  | France |
| GR–DEU |  |  | Germany |
| LI–LVA | Linseed | Cold pressed | Latvia |
| LI–POL |  |  | Poland |
| LI–DEU |  |  | Germany |
| LI–ITA |  |  | Italy |
| RA–LVA | Rapeseed | Cold pressed | Latvia |
| RA–POL |  |  | Poland |
| RA–SWE |  |  | Sweden |
| RA–DEU |  |  | Germany |
| OL–ITA | Olive | Extra virgin | Italy |
| OL–ESP |  |  | Spain |
| OL–PRT |  |  | Portugal |
| OL–GRC |  |  | Greece |
| MI–LVA | Milk thistle | Cold pressed | Latvia |
| MI–POL |  |  | Poland |
| MI–SVN |  |  | Slovenia |
| MI–DEU |  |  | Germany |
| RI–ITA | Rice bran | Extracted | Italy |
| RI–DEU |  |  | Germany |
| RI–IND |  |  | India |
| RI–USA |  |  | United States |
| SU–POL | Sunflower | Cold pressed | Poland |
| SU–UKR |  |  | Ukraine |
| SU–DEU |  |  | Germany |
| SU–ITA |  |  | Italy |

Supplementary Table S2.

Radical scavenging activity values obtained by UV/VIS spectrophotometry and smartphone image analysis using individual and combined RGB colour-channel responses.

| Vegetable oils /  Sample code | | Method | | | | | | | | | | |
| --- | --- | --- | --- | --- | --- | --- | --- | --- | --- | --- | --- | --- |
|  |  | UV-VIS | Smartphone image analysis | | | | | | | | | |
|  |  | Radical scavenging activity (RSA), % | | | | | | | | | | |
|  |  |  | R | G | | B | RG | | RB | GB | | RGB |
| Sea buckthorn | SE-LVA | 93.5 ± 0.3 | 94.8 ± 0.7 | 91.7 ± 0.7 | 67.0 ± 0.6 | | 93.2 ± 0.7 | 80.9 ± 0.6 | | 79.3 ± 0.6 | 84.5 ± 0.6 | |
|  | SE-EST | 85.0 ± 0.3 | 86.1 ± 0.7 | 83.3 ± 0.7 | 61.4 ± 0.6 | | 85.1 ± 0.7 | 73.8 ± 0.6 | | 72.1 ± 0.6 | 76.9 ± 0.6 | |
|  | SE-POL | 80.8 ± 0.2 | 81.5 ± 0.7 | 79.2 ± 0.7 | 58.3 ± 0.6 | | 80.6 ± 0.7 | 69.8 ± 0.6 | | 68.3 ± 0.6 | 72.8 ± 0.6 | |
|  | SE-DEU | 76.2 ± 0.2 | 77.1 ± 0.7 | 74.7 ± 0.7 | 55.0 ± 0.6 | | 75.7 ± 0.7 | 65.8 ± 0.6 | | 64.2 ± 0.6 | 68.6 ± 0.6 | |
| Sunflower | SU-DEU | 80.7 ± 0.2 | 85.2 ± 0.6 | 76.1 ± 0.6 | 40.6 ± 0.6 | | 80.7 ± 0.6 | 62.9 ± 0.6 | | 58.4 ± 0.6 | 67.3 ± 0.6 | |
|  | SU-POL | 74.3 ± 0.2 | 78.4 ± 0.6 | 70.0 ± 0.6 | 37.3 ± 0.6 | | 73.8 ± 0.6 | 58.1 ± 0.6 | | 54.0 ± 0.6 | 62.2 ± 0.6 | |
|  | SU-UKR | 85.0 ± 0.3 | 89.6 ± 0.6 | 79.3 ± 0.6 | 37.6 ± 0.6 | | 85.8 ± 0.6 | 64.4 ± 0.6 | | 79.2 ± 0.6 | 69.6 ± 0.6 | |
|  | SU-ITA | 77.1 ± 0.2 | 81.4 ± 0.6 | 72.7 ± 0.6 | 38.7 ± 0.6 | | 77.3 ± 0.6 | 60.4 ± 0.6 | | 56.2 ± 0.6 | 64.6 ± 0.6 | |
| Rice bran | RI-ITA | 67.6 ± 0.2 | 74.8 ± 0.7 | 59.5 ± 0.7 | 0.9 ± 0.6 | | 67.1 ± 0.6 | 37.9 ± 0.6 | | 30.2 ± 0.6 | 45.0 ± 0.7 | |
|  | RI-DEU | 73.0 ± 0.2 | 80.8 ± 0.7 | 64.3 ± 0.7 | 1.0 ± 0.6 | | 72.8 ± 0.6 | 41.0 ± 0.6 | | 32.7 ± 0.6 | 48.7 ± 0.7 | |
|  | RI-IND | 77.0 ± 0.2 | 85.3 ± 0.7 | 67.9 ± 0.7 | 1.1 ± 0.6 | | 77.3 ± 0.6 | 43.3 ± 0.6 | | 34.5 ± 0.6 | 51.5 ± 0.7 | |
|  | RI-USA | 75.2 ± 0.2 | 83.3 ± 0.7 | 66.3 ± 0.7 | 1.0 ± 0.6 | | 74.8 ± 0.6 | 42.2 ± 0.6 | | 33.7 ± 0.6 | 50.4 ± 0.7 | |
| Hemp | HE-LVA | 95.7 ± 0.3 | 96.0 ± 0.7 | 95.0 ± 0.7 | 68.1 ± 0.6 | | 95.5 ± 0.7 | 82.0 ± 0.6 | | 82.0 ± 0.6 | 86.4 ± 0.6 | |
|  | HE-LTU | 89.4 ± 0.3 | 89.8 ± 0.7 | 88.9 ± 0.7 | 63.8 ± 0.6 | | 89.5 ± 0.7 | 76.9 ± 0.6 | | 76.7 ± 0.6 | 80.3 ± 0.6 | |
|  | HE-POL | 84.1 ± 0.2 | 84.5 ± 0.7 | 83.6 ± 0.7 | 59.9 ± 0.6 | | 84.0 ± 0.7 | 72.1 ± 0.6 | | 72.0±0.6 | 75.4 ± 0.6 | |
|  | HE- DEU | 81.2 ± 0.2 | 81.6 ± 0.7 | 80.7 ± 0.7 | 57.6 ± 0.6 | | 81.0 ± 0.7 | 69.5 ± 0.6 | | 69.3 ± 0.6 | 72.5 ± 0.6 | |
| Corn | CO-ITA | 61.5 ± 0.2 | 74.8 ± 0.6 | 47.9 ± 0.8 | 1.3 ± 0.6 | | 61.3 ± 0.6 | 38.0 ± 0.6 | | 24.6 ± 0.6 | 41.3 ± 0.6 | |
|  | CO-HUN | 71.6 ± 0.2 | 87.1 ± 0.6 | 55.7 ± 0.8 | 1.5 ± 0.6 | | 71.3 ± 0.6 | 44.3 ± 0.6 | | 28.7 ± 0.6 | 48.2 ± 0.6 | |
|  | CO-POL | 68.2 ± 0.2 | 83.0 ± 0.6 | 53.1 ± 0.8 | 1.4 ± 0.6 | | 67.7 ± 0.6 | 42.2 ± 0.6 | | 27.3 ± 0.6 | 45.9 ± 0.6 | |
|  | CO-UKR | 77.3 ± 0.2 | 94.2 ± 0.6 | 60.3 ± 0.8 | 1.6 ± 0.6 | | 76.7 ± 0.6 | 47.8 ± 0.6 | | 30.9 ± 0.6 | 51.8 ± 0.6 | |
| Grape | GR- ITA | 73.3 ± 0.2 | 84.2 ± 0.6 | 61.6 ± 0.6 | 34.4 ± 0.6 | | 72.9 ± 0.6 | 59.3 ± 0.6 | | 48.0 ± 0.6 | 43.4 ± 0.6 | |
|  | GR-ESP | 88.7 ± 0.3 | 97.8 ± 0.6 | 71.6 ± 0.6 | 40.1 ± 0.6 | | 85.7 ± 0.6 | 67.6 ± 0.6 | | 54.8±0.6 | 49.5±0.6 | |
|  | GR-FRA | 59.1 ± 0.2 | 67.9±0.6 | 49.7 ± 0.6 | 27.9 ± 0.6 | | 56.7 ± 0.6 | 45.8 ± 0.6 | | 37.2±0.6 | 33.6±0.6 | |
|  | GR-DEU | 66.2 ± 0.2 | 76.1 ± 0.6 | 55.7 ± 0.6 | 31.3 ± 0.6 | | 63.3 ± 0.6 | 51.2 ± 0.6 | | 41.5±0.6 | 37.6±0.6 | |
| Linseed | LI-ITA | 68.3 ± 0.2 | 77.4 ± 0.6 | 59.5 ± 0.6 | 15.3 ± 0.6 | | 68.5±0.6 | 46.4±0.6 | | 37.4±0.6 | 52.3±0.6 | |
|  | LI-POL | 66.5 ± 0.2 | 75.4 ± 0.6 | 58.0 ± 0.6 | 14.9 ± 0.6 | | 66.8±0.6 | 45.1±0.6 | | 36.3±0.6 | 50.7±0.6 | |
|  | LI-DEU | 64.0 ± 0.2 | 72.5 ± 0.6 | 55.8 ± 0.6 | 14.3 ± 0.6 | | 63.6±0.6 | 43.4±0.6 | | 35.0±0.6 | 49.2±0.6 | |
|  | LI-LVA | 72.0 ± 0.2 | 81.6 ± 0.6 | 62.8 ± 0.6 | 16.2 ± 0.6 | | 72.5±0.6 | 49.0±0.6 | | 39.5±0.6 | 55.0±0.6 | |
| Rapeseed | RA-LVA | 91.4 ± 0.3 | 89.7 ± 0.6 | 93.6 ± 0.7 | 46.1 ± 0.6 | | 91.7±0.7 | 67.9±0.6 | | 69.9±0.6 | 76.5±0.6 | |
|  | RA-POL | 87.6 ± 0.3 | 86.2 ± 0.6 | 90.0 ± 0.7 | 44.2 ± 0.6 | | 87.5±0.7 | 64.7±0.6 | | 66.5±0.6 | 72.7±0.6 | |
|  | RA-SWE | 95.2 ± 0.3 | 94.2 ± 0.6 | 97.4 ± 0.7 | 46.1 ± 0.6 | | 94.9±0.7 | 69.4±0.6 | | 71.6±0.6 | 71.6±0.6 | |
|  | RA-DEU | 81.0 ± 0.3 | 79.6 ± 0.6 | 83.1 ± 0.7 | 41.0 ± 0.6 | | 80.7±0.7 | 59.8±0.6 | | 61.5±0.6 | 67.4±0.6 | |
| Olive | OL-ITA | 95.7 ± 0.3 | 96.0 ± 0.7 | 95.0 ± 0.7 | 68.1 ± 0.6 | | 95.5±0.7 | 82.0±0.6 | | 82.0±0.6 | 86.4±0.6 | |
|  | OL-ESP | 96.0 ± 0.3 | 96.2 ± 0.7 | 95.2 ± 0.7 | 70.0 ± 0.6 | | 96.3±0.7 | 83.1±0.6 | | 82.9±0.6 | 87.3±0.6 | |
|  | OL-PRT | 97.5 ± 0.3 | 97.9 ± 0.7 | 96.5 ± 0.7 | 72.2 ± 0.6 | | 97.2±0.7 | 84.0±0.6 | | 84.5±0.6 | 89.1±0.6 | |
|  | OL-GRC | 96.8 ± 0.3 | 97.7 ± 0.7 | 96.0 ± 0.7 | 71.5 ± 0.6 | | 96.4±0.7 | 84.4±0.6 | | 83.8±0.6 | 88.5±0.6 | |
| Milk thistle | MI-LVA | 94.3 ± 0.3 | 96.9 ± 0.6 | 92.2 ± 0.6 | 80.0 ± 0.7 | | 94.6 ± 0.6 | 88.5 ± 0.6 | | 86.1 ± 0.6 | 89.7 ± 0.6 | |
|  | MI-POL | 84.2 ± 0.3 | 86.5 ± 0.6 | 82.3 ± 0.6 | 71.3 ± 0.7 | | 84.7 ± 0.6 | 79.0 ± 0.6 | | 76.8 ± 0.6 | 80.0 ± 0.6 | |
|  | MI-SVN | 89.5 ± 0.3 | 91.8 ± 0.6 | 87.3 ± 0.6 | 75.6 ± 0.7 | | 89.9 ± 0.6 | 84.1 ± 0.6 | | 81.8 ± 0.6 | 85.2 ± 0.6 | |
|  | MI-DEU | 78.7 ± 0.2 | 80.3 ± 0.6 | 76.3 ± 0.6 | 66.1 ± 0.7 | | 81.3 ± 0.6 | 74.0 ± 0.6 | | 71.8 ± 0.6 | 75.0 ± 0.6 | |

Values are expressed as mean ± standard deviation of ten repeated signal acquisitions from the same prepared solution in the same cuvette (*n*=10). These repetitions represent instrumental-reading and image-acquisition repeatability and do not represent independent sample preparations. R, G and B denote individual colour-channel responses; RG, RB, GB and RGB denote combined colour-channel responses.
